# A transgenic zebrafish model for *in vivo* long-term imaging of retinotectal synaptogenesis

**DOI:** 10.1038/s41598-018-32409-y

**Published:** 2018-09-19

**Authors:** Xu-fei Du, Bing Xu, Yu Zhang, Min-jia Chen, Jiu-lin Du

**Affiliations:** 1 Institute of Neuroscience, State Key Laboratory of Neuroscience, Center for Excellence in Brain Science and Intelligence Technology, Chinese Academy of Sciences, 320 Yue-Yang Road, Shanghai, 200031 China; 2 School of Future Technology, University of Chinese Academy of Sciences, 19A Yu-Quan Road, Beijing, 100049 China; 3 School of Life Science and Technology, ShanghaiTech University, 319 Yue-Yang Road, Shanghai, 200031 China

## Abstract

The retinotectal synapse in larval zebrafish, combined with live time-lapse imaging, provides an advantageous model for study of the development and remodelling of central synapses *in vivo*. In previous studies, these synapses were labelled by transient expression of fluorescence-tagged synaptic proteins, which resulted in the dramatic variation of labelling patterns in each larva. Here, using GAL4-Upstream Activating Sequence (GAL4-UAS) methodology, we generated stable transgenic lines, which express EGFP-tagged synaptophysin (a presynaptic protein) in retinal ganglion cells (RGCs), to reliably label the pre-synaptic site of retinotectal synapses. This tool avoids the variable labelling of RGCs that occurs in transient transgenic larvae. We obtained several stable transgenic lines that differ consistently in the number of labelled RGCs. Using stable lines that consistently had a single labelled RGC, we could trace synaptogenic dynamics on an individual RGC axonal arbor across different developmental stages. In the stable lines that consistently had multiple labelled RGCs, we could simultaneously monitor both pre- and post-synaptic compartments by combining transient labelling of post-synaptic sites on individual tectal neurons. These tools allowed us to investigate molecular events underlying synaptogenesis and found that the *microRNA-132* (*miR-132*) is required for developmental synaptogenesis. Thus, these transgenic zebrafish stable lines provide appropriate tools for studying central synaptogenesis and underlying molecular mechanisms in intact vertebrate brain.

## Introduction

Developmental synaptogenesis is a fundamental process for building neural circuits in the brain^1–3^. *In vivo* imaging of synaptic molecules fused to fluorescent proteins in specific neuronal populations is indispensable for studying the spatial and temporal dynamics synaptogenesis at both cellular and molecular levels^4–6^. Among *in vivo* vertebrate models for imaging synaptogenesis, the retinotectal synapse in larval zebrafish is an excellent system in particular for several reasons: (1) it is a central synapse formed between retinal ganglion cells (RGCs) and tectal neurons; (2) genetic tools are available for visualizing this synapse; (3) larval zebrafish are transparent and this synapse locates in the optic tectum, the most dorsal brain area in fish, and thus is accessible to live imaging^7^; (4) the visual system of zebrafish develops rapidly^8,9^, making it possible to capture the whole process of visual connection formation in intact animals; (5) zebrafish is amenable to both forward and reverse genetic manipulations for dissection of molecular mechanisms underlying synaptogenesis.

To visualize retinotectal synapses, Stephen Smith’s laboratory developed GAL4-Upstream Activating Sequence (GAL4-UAS) binary tools and microinjected them into embryos at the one-cell stage to transiently express fluorescence-tagged pre- and post-synaptic marker proteins in RGCs and tectal neurons, respectively^10,11^. Transient expression can lead to mosaic labelling of neurons, allowing imaging of synaptogenic dynamics on axonal or dendritic arbors of individually resolvable neurons over successive days^10–13^. However, as the labelling stability and efficiency of transient expression is relatively low, the transient strategy is mainly applicable to relative short-term imaging and inefficient for investigating molecular mechanisms through forward and reverse genetic manipulations. Here, using GAL4-UAS binary tools for labelling pre-synaptic terminals of RGCs, we generated double transgenic zebrafish stable lines for *in vivo* long-term imaging of retinotectal synaptogenesis. We first tested the reliability of the transgenic labelling for long-term time-lapse imaging with temporal resolution at different scales. We further applied this model to monitor both pre- and post-synaptic markers simultaneously and examined the potential role of a specific microRNA (miRNA) in developmental synaptogenesis.

MicroRNAs are small, non-coding RNAs that mediate post-transcriptional gene regulation^14–16^. Several *in vitro* studies revealed that *miR-132*, a CREB- (cAMP-response element binding) and neuronal activity-regulated miRNA, is involved in dendritogenesis and spinogenesis by targeting the GTPase-activating protein p250GAP^17–19^. It is also found that *miR-132* plays regulatory roles in dendritic spine plasticity^20,21^ and experience-dependent ocular dominance plasticity^22,23^. However, whether *miR-132* affects synaptogenesis during development remains unclear. Using *in vivo* long-term imaging on stable transgenic zebrafish lines, in which retinotectal synapses were fluorescently labelled, we provide *in vivo* evidence for the role of *miR-132* in regulating developmental synaptogenesis.

## Results

### Visualizing retinotectal synapses in double transgenic zebrafish larvae

**Figure 1:**
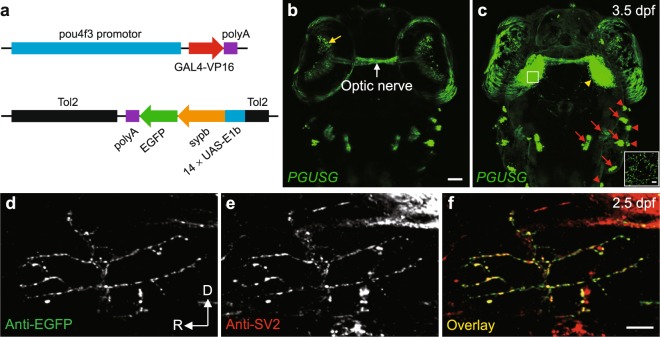
Visualization of RGC pre-synaptic terminals in double transgenic zebrafish *PGUSG*. (**a**) Schematic of the DNA constructs used for generating the GAL4 line *Tg(pou4f3:GAL4-VP16)* (*PG*) and the UAS line *Tg(14* × *UAS-E1b:sypb-EGFP)* (*USG*). (**b**,**c**) Dorsal views of a *PGUSG* double transgenic larva at 3.5 dpf showing EGFP expression in RGCs (soma in the retina, yellow arrow; axonal arbors in the tectal neuropil, yellow arrow head) and mechanosensory hair cells of the inner ear (red arrow) and lateral line (red arrow head). Images in (**b**) and (**c**) are the maximum-intensity-projection view of more ventral z-stack and the whole z-stack, respectively. Inset in (**c**), zoom-in view of a single optical section of the boxed area in left tectal neuropil showing the punctate signal of Sypb-EGFP on RGC axonal arbor. The optic nerve (white arrow) is visible. The nasal is upwards. Scale bar, 50 μm for each panel; 5 μm for inset. (**d–f**) Confocal images of single spinal motor neuron in *EGUSG* (see Methods) double immuno-labelled with EGFP (**d**) and SV2 (**e**) antibodies at 2.5 dpf. Colocalization of Sypb-EGFP with pre-synaptic SV2 is demonstrated by the overlap (**f**, yellow) of anti-EGFP and anti-SV2 puncta. D, dorsal; R, rostral. Scale bar, 10 μm.

To generate GAL4-UAS double transgenic zebrafish for *in vivo* imaging of retinotectal synaptogenesis, we first created the GAL4 and UAS transgenic lines, which carry the activator and effector constructs, respectively. In activator lines, the *pou4f3* (also known as *brn3c*) promotor drives the transcriptional activator *GAL4-VP16* (Fig. 1a, top). And in effector lines, the DNA-binding motif of GAL4, 14 × *UAS-E1b*, controls the zebrafish synaptic vesicle protein *synaptophysin b* (*sypb*, also known as *Syp* in mammals) fused with *enhanced green fluorescent protein* (*EGFP*), and the whole construct^10^ is flanked by *Tol2 cis* sequences for transposition^24^ (Fig. 1a, bottom) (see also Methods). Crossing the activator and effector lines gave rise to the double transgenic fish *Tg(pou4f3:GAL4-VP16)*^*ion6d*^*;Tg(14* × *UAS-E1b:sypb-EGFP)*^*ion7d*^ (*PGUSG*), in which the effector gene is expressed in a spatial pattern as that of the activator controlled by *pou4f3* promotor^25^, including a subset of RGCs, and mechanosensory hair cells in the inner ear and lateral line (Fig. 1b,c). The Sypb-EGFP signal on RGC axonal arbors in the tectal neuropil showed a punctate structure (Fig. 1c, inset).

Synaptophysin fused to EGFP is a validated pre-synaptic marker for live imaging of synapses in animals with transient expression of the fused protein^10,13,26,27^. To validate whether Sypb-EGFP puncta in stable transgenic lines well mark pre-synaptic sites, we evaluated its colocalization with the synaptic vesicle protein 2 (SV2) by whole-mount double immunohistochemistry (IHC) with antibodies against EGFP and SV2. We examined the degree of colocalization in the developing zebrafish spinal cord where SV2 immunostaining was less dense. To achieve this, we generated another double transgenic stable line *Tg(elavl3:GAL4-VP16)*^*ion8d*^*; Tg(14* × *UAS-E1b:sypb-EGFP)*^*ion7d*^ (*EGUSG*), in which the spinal motor neurons could be sparsely labelled by Sypb-EGFP. In 2.5-dpf *EGUSG* larvae, we scored the percentage of anti-EGFP puncta (Fig. 1d) on the individual spinal motor neuron that colocalized with anti-SV2 puncta (Fig. 1e) and found a high colocalization probability (Fig. 1f; 87.6 ± 2.5%, n = 11; see also Methods). These data show that Sypb-EGFP puncta in transgenic larvae largely correspond to pre-synaptic sites on neuronal axons in zebrafish.

### Diverse labelling patterns of RGCs in double transgenic zebrafish

**Figure 2:**
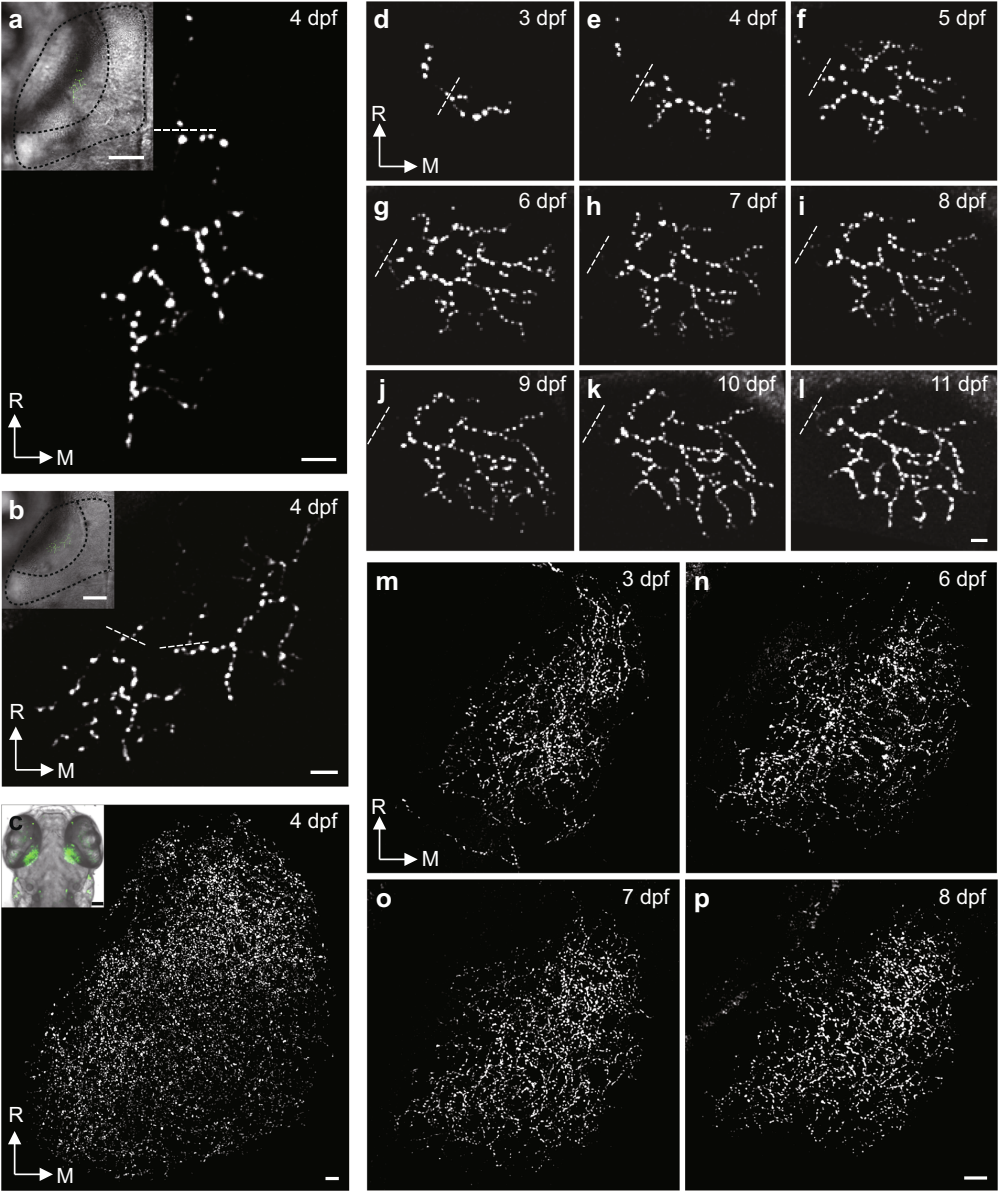
Long-term stable *in vivo* imaging of retinotectal pre-synaptic terminals in *PGUSG* larvae with single or multiple RGCs labelled. (**a**–**c**) Examples of single (**a**), two (**b**) and multiple (**c**) Sypb-EGFP labelled RGC axonal arbors within one tectal hemisphere from different *PGUSG* fish at 4 dpf. Insets, topographic position of RGC axonal arbors in the tectal neuropil. In each inset, an optical section of bright-field image and a stack of green fluorescent images are superimposed. Dashed black curve, the profiles of half tectum and half neuropil. (**d–p**) Time series showing maximum-intensity-projection view of the same single (**d–l**) and multiple (**m–p**) Sypb-EGFP labelled RGC axonal arbors at 3–11 dpf and at 3, 6, 7 and 8 dpf, respectively. Autofluorescence of the skin is visible in the right upper corner in panel (**j**), (**k**) and (**l**). Dashed white line, position above the first branch point of each axonal arbor. R, rostral; M, medial. Scale bar: 5 μm in each panel; 50 μm in panel inset.

Interestingly, due to the position-effect variegation of GAL4-UAS transgenes^28^, the labelling pattern of RGC axonal arbors within the tectum is strikingly diverse in different *PGUSG* stable lines, embodied by the number and the topographic position of axonal arbors and the expression level of Sypb-EGFP. In one tectal hemisphere of *PGUSG* lines, the number of labelled RGC axonal arbors ranged from only one to ~ 30% of *pou4f3*-positive RGCs (Supplementary Fig. S1). Even with the same number of labelled RGCs, the topographic position of the axonal arbors in the tectum was variable cross cells and cross fish. Fig. 2 shows examples of punctate Sypb-EGFP expression on an individual (Fig. 2a), two topographically separate (Fig. 2b), and multiple RGC axonal arbors that cover the entire tectal neuropil in the maximum projection view (Fig. 2c). This variability could be found among larvae from different lines, and each stable double transgenic line had its characteristic labelling number of RGCs.

To investigate whether the labelling pattern is preserved throughout development, we selected *PGUSG* larvae with one or several labelled RGCs’ axonal arbor in one tectal hemisphere and performed two-photon time-lapse imaging for consecutive days from 3 to 11 dpf. We found no increase in the number of labelled RGC axonal arbors during development, indicating that once the expression pattern is determined at the time (70–72 hpf) when retinal axons reach the tectal neuropil^29^, it persists through development. Thus we could follow the development of pre-synaptic sites in transgenic fish with a single labelled RGC axonal arbor (Fig. 2d–l) as well as those with a population of labelled RGC axonal arbors (Fig. 2m–p). Therefore, *PGUSG* offers a reliable model for studying central synaptogenesis *in vivo*.

### Long-term imaging of retinotectal synapse remodelling in larval zebrafish

**Figure 3:**
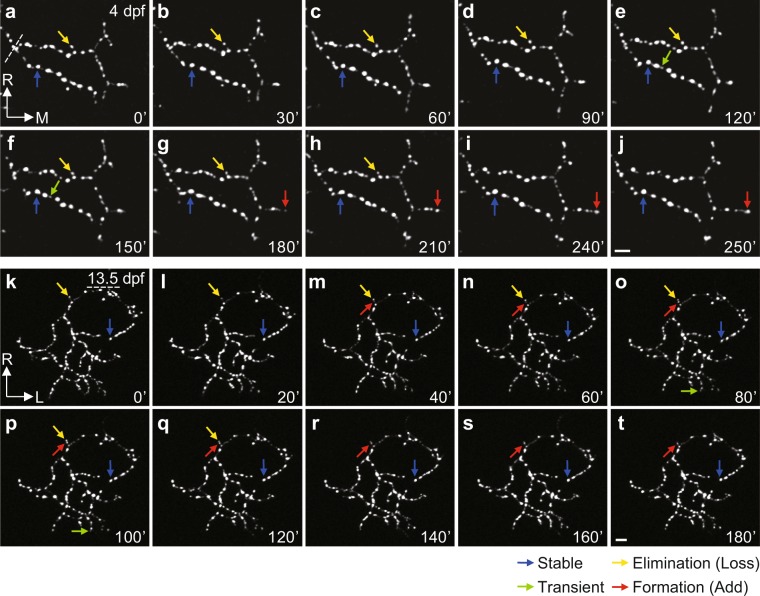
Remodelling of Sypb-EGFP puncta on the axonal arbor of individual RGCs. (**a–t**) Time-lapse two-photon images of individual RGC axonal arbor expressing Sypb-EGFP showing synaptic remodelling at both early (4 dpf, **a–j**) and late (13.5 dpf, **k–t**) larval stages, respectively. At 13.5 dpf, we used pigment mutants of *PGUSG* (*PGUSG*; *casper*). Arbors were imaged at 10 min intervals for at least 3 h. Time in minutes is indicated in the bottom right corner of each panel. The coloured arrows indicate examples of puncta with different behaviours: stable (blue), existing through the entire imaging time period; elimination (yellow), existing at the beginning but lost during imaging; transient (green), newly added during imaging but lost before the end of imaging; formation (red), newly added during imaging and maintained till the end of imaging. L, lateral; M, medial; R, rostral. Scale bar, 5 μm.

*In vivo* time-lapse imaging reveals that the construction of neuronal connections is a dynamic process consisting of the concurrent formation and elimination of arbors and synapses^10–13,30^. To determine whether *PGUSG* is a good model for studying the synaptogenic dynamics, we performed two-photon time-lapse imaging at a 10-min interval on the same individual RGC axonal arbor expressing Sypb-EGFP (Fig. 3a–j). We found that during 4–5 dpf, RGC axonal arbors added 182 ± 8.6% and lose 175 ± 7.6% of the initial number of Sypb-EGFP puncta within 4 h, yielding a synaptic turnover rate (N_formation_ + N_elimination_/2N_total_) as 16 ± 0.3% per hour (n = 20). The higher rate of synapse formation leads to a gradual increase of synapse number during development. These results further demonstrate that central synaptogenesis is a highly dynamic process consisting of rapid and nearly balanced synapse formation and elimination. Thus the *PGUSG* larvae with a single labelled RGC provide a suitable model for quantitatively analysing synaptogenic dynamics on individual central neurons in the developing brain.

The zebrafish visual system develops rapidly^8,9^. After 7 dpf, the total length of RGC axonal arbors and tectal cell dendrites as well as the number of synaptic puncta on them remain relatively stable^10,11^. Thus the visual system of larvae older than one week reaches a relatively mature state. In the mature brain, the central synapse is known to undergo structural remodelling to support functional changes. Furthermore, as teleost fish grow continuously throughout their lifespan^31^, the retinotectal synapse must continuously undergo structural changes to coordinate growth between the retina and optic tectum. To observe structural synaptic remodelling on individual RGC axonal arbors in older larvae, we generated the *PGUSG; casper* line, which is transparent throughout lifespan^32^. We selected *PGUSG* fish that showed bright labelling on a single RGC axonal arbor at 7 dpf and performed time-lapse imaging with a 10-min interval at ~14 dpf. We observed the formation and elimination of Sypb-EGFP puncta on branches at all orders of the axonal arbor without changes in gross morphology or synapse number (Fig. 3k–t). This optically transparent *PGUSG; casper* can be used as a model for studying retinotectal structural plasticity and remodelling in the intact mature brain.

### Simultaneous imaging of both pre- and post-synaptic compartments *in vivo*

Synaptogenesis is a complex process which requires spatially and temporally coordinated development and interaction between pre- and post-synaptic compartments^1,2^. Unlike neuromuscular junctions, neuron-neuron synaptic contacts in the central nervous system have been far less accessible due to the technical challenge in simultaneous labelling of both pre- and post-synaptic partners of single synapses. The *PGUSG* larvae with the Sypb-EGFP expression on a large number of RGC axonal arbors may provide us with an opportunity to image interneuronal synaptogenesis *in vivo*.

**Figure 4:**
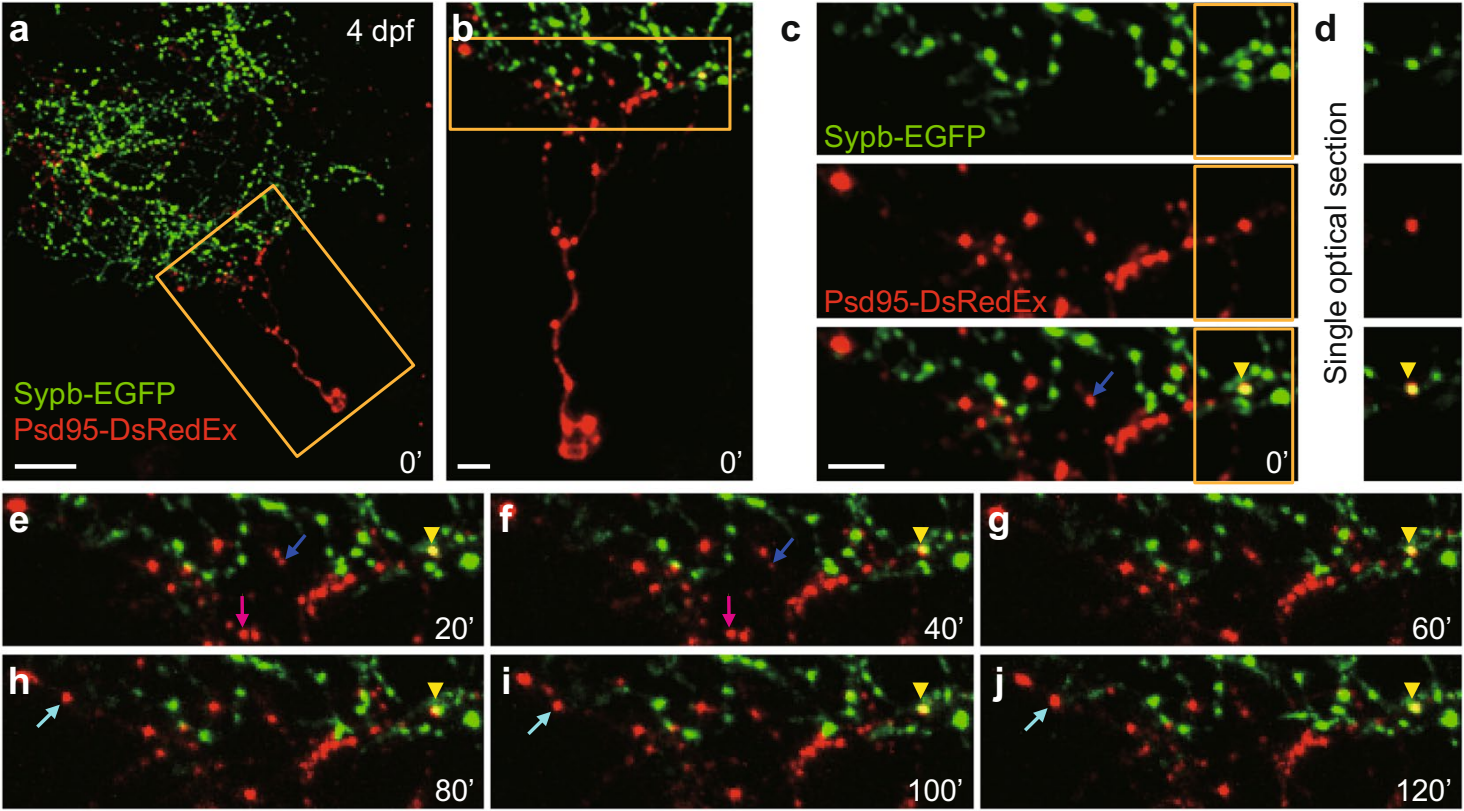
Simultaneous imaging of pre- and post-synaptic compartments of retinotectal synapses. (**a**) First image of a time-lapse series showing the Psd95-DsRedEx labelled post-synaptic sites on the dendritic arbor of a tectal neuron (red) and the Sypb-EGFP labelled pre-synaptic terminals on the axonal arbor of multiple RGCs (green) in a 4-dpf *PGUSG* larva with transient expression of *elavl3:psd95-DsRedEx*. The nasal is upwards. Scale bar, 20 μm. (**b**) Enlarged view of the boxed region in. (**a**) Scale bar, 5 μm. (**c**) Enlarged view of the boxed region in (**b**) showing single-channel images and the composite image. The yellow arrowhead (bottom) indicates the association of a pre-synaptic terminal labelled by Sypb-EGFP (green, top) with a post-synaptic site labelled by Psd95-DsRedEx (red, middle). (**d**) Single optical section of the boxed region in (**c**) showing the fluorescence overlap of the pre- and post-synaptic punctum. (**c**,**e–j**) 2-h time series with a 20-min interval showing the overlap of pre- and post-synaptic labels (yellow arrowheads) is stably maintained. Time in minutes is indicated at the bottom right corner of each panel. Postsynaptic Psd95-DsRedEx puncta also showed dynamic behaviours: elimination (blue arrow), transient (magenta arrow), and formation (cyan arrow). Scale bar, 5 μm.

To visualize retinotectal synaptic contacts in the *PGUSG* larvae with multiple RGCs labelled, we sparsely labelled single tectal neurons with the fluorescence-tagged post-synaptic marker protein Psd95-DsRedEx which is driven by the pan-neuronal *elavl3* (also known as *HuC*) promotor^33^ (Fig. 4a). We first validated that the Psd95-DsRedEx puncta indeed mark the post-synaptic sites by showing the overlapping juxtaposition of DsRed and SV2 immuno-labelled puncta in the tectal neuropil (Supplementary Fig. S2 and Methods). Transient expression of Psd95-DsRedEx in *PGUSG* larvae allows us to simultaneously visualize both pre- and post-synaptic structures of retinotectal synapses. Fig. 4 shows an example of simultaneous time-lapse imaging of pre-synaptic Sypb-EGFP puncta and post-synaptic Psd95-DsRedEx puncta with a 20-min interval for 2 h at 4 dpf (see also Supplementary Fig. S3). Analysis of the time-lapse sequences revealed synaptic dynamics for both pre- and post-synaptic sites (Fig. 4c,e–j, coloured arrow). We observed stably maintained associations of pre- and post-synaptic puncta (Fig. 4c–j; Supplementary Fig. S3c–i, yellow arrowhead), implying the putative synaptic contacts, but additional functional or ultrastructural examination is still needed to further confirm the synapse identity. These data suggest that it is possible to use *PGUSG* larvae expressing *elavl3:Psd95-DsRedEx* to study central synaptogenesis by long-term monitoring of both pre- and post-synaptic structures *in vivo*.

### Essential role of *miR-132* in developmental retinotectal synaptogenesis

**Figure 5:**
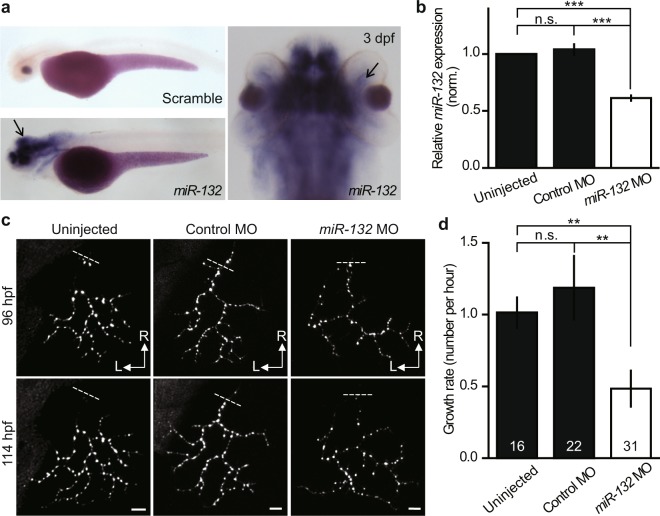
Role of *miR-132* in developmental synaptogenesis. (**a**) Lateral (left bottom) and dorsal (right) view of whole-mount *in situ* hybridization of *miR-132* in zebrafish larvae at 3 dpf. The black arrows in left and right panels indicate the signal in the optic tectum and the RGC layer, respectively. Left top, whole-mount *in situ* hybridization of a scramble probe as a control. (**b**) Knockdown efficiency of *miR-132* MO. Quantification of mature *miR-132* in 3-dpf zebrafish larvae without MO injection or injected with either control or *miR-132* MO by relative quantitative real-time PCR. Data were summarized from six independent experiments. n.s., no significant, ***P < 0.001 (One-way ANOVA and Tukey’s multiple comparison test). (**c**) Example of time series (from 96 hpf to 114 hpf) images showing the growth of Sypb-EGFP puncta on the same RGC axonal arbors in *PGUSG* larvae without MO injection or injected with either control or *miR-132* MO. The dashed line indicates the position of the first branch point in each axonal arbor. R, rostral; L, lateral. Scale bar, 5 μm. (**d**) Summary of the net growth rate of Sypb-EGFP puncta from 96 hpf to 114 hpf. The numbers on the bars indicate the number of RGCs examined. n.s., no significant, **P < 0.01 (Two-tailed Unpaired Student’s *t*-test).

In addition to the observation of synaptogenesis by live optical imaging, it is also important to investigate molecular mechanisms underlying synaptogenesis through forward and reverse genetic manipulations. Using *PGUSG* larvae carrying a single labelled RGC, we examined whether *miR-132*, a small non-coding RNA, plays roles in developmental synaptogenesis. *MiR-132* is a brain-enriched miRNA and has been found to be involved in dendritogenesis and spinogenesis *in vitro*^17–19^. Whole-mount *in situ* of *miR-132* showed that it expresses in both the optic tectum and the RGC layer (Fig. 5a). To manipulate the expression of *miR-132*, we microinjected antisense morpholino oligomers (MO) specific for mature *miR-132*^34,35^ into *PGUSG* embryos at the one-cell stage to reduce the *miR-132* level. To determine the knockdown efficiency and specificity of *miR-132* MO^36^, we quantified the amount of *miR-132* and another brain-enriched *miR-219*^37^ by quantitative real-time PCR (qRT-PCR). In *miR-132* morphants, the expression level of *miR-132* was significantly reduced (61.2 ± 2.4%, n = 6, *P* < 0.001) in comparison with control fish (Fig. 5b), but the expression of *miR-219* was not affected (Supplementary Fig. S4). We then performed time-lapse imaging on single RGC axonal arbors at 96 hpf and 114 hpf (Fig. 5c) and measured the change of the total number of Sypb-EGFP puncta on the same axonal arbors. We found that during 96–114 hpf, the net growth rate of puncta was significantly slower in *miR-132* morphants than in control fish (Fig. 5d; *P* < 0.01), indicating *miR-132* is involved in the development of central synaptogenesis *in vivo*.

## Discussion

In this study, we created the double transgenic zebrafish *PGUSG* to visualize retinotectal synapses in intact animals. We found that different *PGUSG* stable lines show different labelling patterns of RGCs, while in individual fish the pattern keeps consistent throughout development. Using *in vivo* two-photon time-lapse imaging of *PGUSG* larvae, we were able to monitor the synapse behaviour on developing RGC axonal arbors over hours, days or even weeks. We then developed methods to simultaneously image both pre- and post-synaptic compartments at putative synaptic connections *in vivo*. Finally, using reverse genetic manipulation on *PGUSG* larvae, we showed that *miR-132* plays a role in developmental synaptogenesis.

Our study showed that the number and topographic position of labelled RGC axonal arbors in *PGUSG* larvae are highly diverse, a phenomenon similar to a previous study in which the GAL4-UAS binary system was used to transgenically express mGFP in RGCs^38^. This diversity may be derived from two sources: (1) the transgene of the activator line. We found that even crossed with the same UAS reporter line, different GAL4-VP16 lines allowed us to label different numbers of RGCs. This variegated (mosaic) expression of UAS-reporters seems to be an intrinsic property in transgenic lines expressing GAL4-VP16 driven by specific promotors that were created by conventional transgenesis^28^; (2) the transgene of the effector line. Although the effector lines were created by *Tol2*-mediated transgenesis, which can lead to consistent transgenic labelling among siblings for GAL4 transgenic lines^28,39^, we found different UAS lines show different labelling patterns when crossed with the same ubiquitously expressed GAL4 line, further supporting the fact that the UAS-transgenes are subjected to position effects^28^. Importantly, despite the variability cross fish and lines, the labelling patterns were consistent throughout development for individual *PGUSG* fish.

As the GAL4-UAS system is a binary system, it brings the flexibility by combining the USG line with other tissue-specific GAL4 lines to study synaptogenesis of other types of neurons at single cell level, or vice versa, by combining the GAL4 line with UAS transgenic lines carrying other reporter genes and effector genes. For instance, the expression of the reporter gene like calcium indicators fused to Sypb would allow simultaneous monitoring of both the structure and function of retinotectal synapses^40^, while the expression of effector gene like the fluorescent protein-tagged tetanus toxin light-chain (TeNT-Lc)^41^, which can block synaptic transmission, would allow us to examine how synaptic activity regulates synaptogenic dynamics on single retinal axons and neighbouring axons under competition^42,43^.

Although synaptic remodelling is a hall mark of early development, synapse formation and elimination are still prevailing in the mature brain to fine-tune neural circuits to adapt to changing environments. To visualize retinotectal synapses in more mature brains of zebrafish, we created *PGUSG;casper*. Taking advantage of its lifelong transparency, we could perform *in vivo* time-lapse imaging of retinotectal synapses on individual RGC axonal arbors in fish of two-weeks old. We found that, at this stage, the axon branching pattern and punctum number are stable, but the punctum turnover still occurs frequently at the timescale of minutes. This is comparable to dynamic dendritic spines and synaptic boutons observed in the cerebral cortex of adult mammals^44,45^.

Besides basal dynamics, *in vivo* studies provide strong evidence for supporting the structural reorganization of synaptic connections during learning and memory^44,46–49^. As it is possible to image synaptic remodelling at high temporal resolution (e.g. 10-min interval) on older *PGUSG; casper* larvae, we can combine behavioural assays^50^ to study how retinotectal synapses change with visual perception-based and behaviour-related experience alteration, learning, and memory in intact larvae.

Synaptogenesis is the formation of cellular junctions between neurons and their targets. We cannot understand the wiring of neuronal circuits without concurrent observing of both pre- and post-synaptic elements. At the mammalian peripheral neuromuscular junction, *in vivo* concurrent imaging of multiple synaptic elements has revealed how multiply innervated junctions transform to singly innervated ones during development^51^. However, due to the technical challenge for *in vivo* imaging of pre- and post-synaptic compartments of identified connections over time during development, details of the formation, maintenance and elimination of central synapses remain largely unknown. One study has attempted to image pre- and post-synaptic sites *in vivo* in the submandibular ganglion in the peripheral nervous system of mice^52^. Here, we found a way of simultaneous imaging of pre and post-synaptic elements of central retinotectal synapses *in vivo* by expressing Psd95-DsRedEx in a single tectal neuron in *PGUSG* larvae with a large population of RGCs labelled by Sypb-EGFP. Although the pre- and post-synaptic markers we used might not be the earliest protein involved in synapse assembly and disassembly, this method allows us to study the dynamic interactions between pre- and post-synaptic elements during synapse formation, stabilization and elimination.

Using MO-mediated gene-specific knockdown, we demonstrated that *miR-132* is important for developmental synaptogenesis. Knockdown of mature *miR-132* significantly reduced the synapse growth rate. As synapse formation and elimination occur concurrently during development, *miR-132* may regulate the rate at which new synapse form and/or the stability of existing synapse. In the future, using time-lapse imaging with high temporal resolution, it is of interest to examine which dynamic processes of synaptogenesis are regulated by *miR-132*.

In mammals, *miR-132* regulates dendrite development and spine formation by inhibiting synthesis of p250GAP^17,18^, which specifically inactivates Rac1 activity in cultured hippocampal neurons^18^. Therefore, it was hypothesized that *miR-132* increases the activity of Rac1, which is a crucial regulator of the actin cytoskeleton, and thus promotes spine growth by suppressing p250GAP^18,53^. In our study, *miR-132* might also regulate retinotectal synaptogenesis by targeting p250GAP. Notably, in our experiments, *miR-132* regulated the growth of pre-synaptic terminals, while p250GAP has been shown to be enriched in the post-synaptic density^54^. So *miR-132* might regulate post-synaptic development and then retrogradely influence pre-synaptic terminals. Alternatively, *miR-132* might directly regulate pre-synaptic terminals by targeting the same or different substrates locating in RGCs.

In sum, the double transgenic zebrafish *PGUSG* is a versatile and reliable model for *in vivo* long-term imaging of retinotectal synapses. We demonstrated the applicability of this model in the long-term imaging of structural synaptic remodelling in both developing and relatively mature brain at the single-cell level, the long-term simultaneous imaging of pre- and post-synaptic elements, and the investigation of molecular mechanisms underlying synapse growth. Therefore, the *PGUSG* provides a transgenic stable model for studying central synaptogenesis in intact vertebrate brains.

## Methods

### Zebrafish lines and maintenance

Adult zebrafish (*Danio rerio*) were maintained in an automatic fish housing system (ESEN, Beijing, China) at 28.5 °C under a 14-h light: 10-h dark cycle following standard protocols. Larval zebrafish were raised in 10% Hank’s solution, which consisted of (in mM) 140 NaCl, 5.4 KCl, 0.25 Na_2_HPO_4_, 0.44 KH_2_PO_4_, 1.3 CaCl_2_, 1.0 MgSO_4_ and 4.2 NaHCO_3_ (pH 7.2), and were treated with 0.003% 1-phenyl-2-thiourea (PTU, Sigma) to prevent pigment formation. The transgenic lines used were as follows (according to ZFIN nomenclature guidelines; abbreviated names are in parentheses):

*Tg(pou4f3:GAL4-VP16)*^*ion6d*^*; Tg(14* × *UAS-E1b:sypb-EGFP)*^*ion7d*^ (*PGUSG*),

*Tg(elavl3:GAL4-VP16)*^*ion8d*^*; Tg(14* × *UAS-E1b:sypb-EGFP)*^*ion7d*^ (*EGUSG*),

*Tg(pou4f3:GAL4-VP16)*^*ion6d*^*; Tg(14* × *UAS-E1b:sypb-EGFP)*^*ion7d*^*; mitfa*^*w2*^*;roy*^*a9*^ (*PGUSG;casper*).

### Generation of DNA constructs and transgenic lines

To generate *Tg(pou4f3:GAL4-VP16);Tg(14* × *UAS-E1b:sypb-EGFP)* (*PGUSG*), the *PG* and *USG* lines were generated independently. For *PG*, *pou4f3:GAL4-VP16* plasmid (gift from Dr. Rachel Wong, University of Washington, Seattle, USA) was linearized with *Afl*II, purified by *Qiaex*II gel extraction kit (Qiagen) and microinjected into embryos at 1–2 cell stage at a concentration of 40 ng/μl. For *USG*, the *Tol2* transposon system^24^ was used. The *USG* fragment was excised with *Dra*III-*Xho*I from *UAS:sypb-EGFP* plasmid^10^ and subcloned into the *Bgl*II-*Xho*I sites in *Tol2-MCS-EGFP* plasmid (gift from Bo Zhang, Peking University, Beijing, China) to replace *MCS-EGFP*. Purified *Tol2-USG* plasmid was microinjected together with *Tol2* transposase mRNA synthesized *in vitro* into embryos at 1–2 cell stage at a concentration of 50 (25 + 25) ng/μl. Injected *PG* and *USG* embryos were raised to sexual maturity and crossed with available UAS and GAL4 line, respectively, to identify founder fish. Founders of *PG* and *USG* were crossed and the embryos from these crosses were scored for their EGFP expression and raised. This led to the production of several *PGUSG* stable lines.

To generate *Tg(elavl3:GAL4-VP16)* (*EG*), the *EG* construct was first generated by subcloning the PCR fragment of *GAL4-VP16* into the *Xho*I-*Xba*I sites of *elavl3:YC2.1* (also known as *HuC:YC2.1*) plasmid^33^ to replace *YC2.1*, and then linearized with *Not*I and microinjected into embryos at 1–2 cell stage.

To generate *elavl3:psd95-DsRedEx* plasmid, zebrafish *psd-95* (*Sal*I-*Sma*I) and *DsRed-Express* (*DsRedEx*) (*Sma*I-*Xba*I) fragments were PCR amplified from adult zebrafish brain cDNA and *pDsRed-Express-1*, respectively, and then subcloned into the *Xho* I-*Xba*I sites of *elavl3:YC2.1*.

### Validation of Sypb-EGFP as a pre-synaptic marker

We first validated transgenic Sypb-EGFP as a pre-synaptic marker by examining the percentage of colocalization of Sypb-EGFP with a known pre-synaptic marker SV2 in developing spinal motor neuron. In transgenic *EGUSG* larvae, the spinal motor neurons can be sparsely labelled by Sypb-EGFP. We performed whole-mount double-IHC on these fish at 2.5 dpf with EGFP and SV2 antibodies. Then we scored the colocalization of anti-EGFP punctum with anti-SV2 punctum by evaluating their overlap (yellow signal) through each optical section in the composite image stack using ImageJ software. And we found 87.6 ± 2.5% of anti-EGFP puncta colocalize with anti-SV2 puncta. Furthermore, based on time-series projections of RGC axons expressing Sypb-EGFP with high temporal resolution (10 min interval), more than 90% of Sypb-EGFP puncta could be distinguished as transporting cluster or not according to their kinetics and we found about 85% were not. Collectively, transgenically expressed Sypb-EGFP puncta on axonal arbors represent pre-synaptic sites in zebrafish.

### Validation of Psd95-DsRedEx as a post-synaptic marker

We also validate Psd95-DsRedEx as a post-synaptic marker by examining its colocalization with the pre-synaptic marker SV2. We fixed fish expressing Psd95-DsRedEx in single or several tectal cells and immuno-labelled them with DsRed and SV2 antibodies on 20–30 μm horizontal cryostat sections at 4 dpf. Due to the high density of anti-SV2 puncta in tectal neuropil, we performed high-resolution confocal imaging of the sections with oil immersion lens (60×) and applied image deconvolution (Huygens Essential) afterwards to further enhance the resolution. We could not find the full overlap of the anti-DsRed and anti-SV2 puncta, but the immediate juxtaposition of the two elements. The juxtaposition is defined as the immunofluorescence intensity overlap >50%, and we found 85.1 ± 1.5% of anti-DsRed puncta juxtapose anti-SV2 puncta. Furthermore, in the tectal neuron, the density of Psd95-DsRedEx puncta along dendrites did not change systematically with distance from the soma. Taken together, the Psd95-DsRedEx puncta are acting as a post-synaptic marker.

### *In vivo* imaging and data analysis

Zebrafish larvae at 3–14 dpf were anesthetized with 0.02% tricaine methanesulfonate (Sigma) or paralyzed with pancuronium dibromide (PCD, 1 mM, Tocris) and then mounted in 1.5% low-melting agarose (Sigma) for imaging, after which the fish were released immediately and reared normally. Two-photon and confocal imaging were performed under 40× (N.A., 0.8) or 20× (N.A., 0.95) water-immersion objective on an upright microscope equipped with a two-photon laser (900 nm) and single-photon lasers at 488 nm and 559 nm, respectively. The step size for z-stack imaging is 1–2 μm.

Punctum counting was performed on maximum intensity projections of image stacks using Image-Pro Plus software. Puncta were identified as discrete local accumulations in Sypb-EGFP of more than 2 native pixels (0.4 μm) in diameter.

### Immunohistochemistry

Phenylthiourea treated and anaesthetized *EGUSG* larvae at 2.5 dpf were fixed in 4% paraformaldehyde (PFA) in phosphate-buffered saline (PBS). To enhance permeability, larvae were incubated in 0.1% trypsin and 0.5% hyaluronidase in PBS for 1 h and 1.5 h, respectively. For section immunostaining, larvae were fixed in 4% PFA and dehydrated with methanol and then embedded for frozen cryostat sectioning. The following antibodies and concentrations were used for whole-mount and frozen section double-IHC: rabbit polyclonal anti-GFP primary antibody (Molecular Probe), 1:800; rabbit polyclonal anti-RFP (including DsRed as the antigen) primary antibody (MBL), 1:500; mouse monoclonal anti-SV2 primary antibody (Hybridoma Bank, Iowa City, IA), 1:50; Alexa Fluor 488 goat-anti-rabbit secondary antibody (Molecular Probe), 1:1000; Alexa Fluor 633 goat-anti-rabbit secondary antibody (Molecular Probe); Alexa Fluor 568 goat-anti-mouse secondary antibody (Molecular Probe), 1:1000.

### Whole-mount *in situ* hybridization

Whole-mount *in situ* hybridization was performed as described previously^35^ with the DIG-labelled *miR-132* LNA probe (5′-CGACCATGGCTGTAGACTGTTA-3′) and the scramble LNA probe (5′-GTGTAACACGTCTATACGCCCA-3′) (Exiqon, Denmark)^34,35^.

### Morpholino knockdown

The *miR-132* morpholino oligomer (MO) targeting the mature *miR-132* in zebrafish (5′-CGACCATGGCTGTAGACTGTTACCA-3′)^35^ and the standard control MO (5′-CCTCTTACCTCAGTTACAATTTATA-3′) were purchased from Gene Tools (Philomath, OR) and microinjected into one-cell-stage embryos at 8 ng.

### Relative quantitative real-time PCR

The total RNA extracted from zebrafish larvae at 3 dpf with Trizol reagent was used to generate cDNA using reverse transcriptase with specific stem–loop primer for miRNA and random primer for other RNA, respectively. Relative quantitative real-time PCR was performed using SYBR Green (Invitrogen) on a Roche LightCycler 480. The relative RNA amount was presented by the comparative C_T_ method with U6 snRNA as internal control. Primers used for real-time and RT-PCR were as follows (5′-3′): GTCGTATCCAGTGCAGGGTCCGAGGTATTCGCACTGGATACGACCGACCA (*miR-132* RT); GTCGTATCCAGTGCAGGGTCCGAGGTATTCGCACTGGATACGACAAGAAT (*miR-219* RT); CGGCGGTAACAGTCTACAGCCA (*miR-132* fwd); CGGCGGTGATTGTCCAAACGCA (*miR-219* fwd); GTGCAGGGTCCGAGGT (*miR-132* & *miR-219* rev); ACTAAAATTGGAACGATACAGAGA (U6 fwd); AAAGATGGAACGCTTCACG (U6 rev).

### Statistical analysis

Statistical analyses were performed as depicted in figure legend using GraphPad Prism (Prism 5) with alpha level set to 0.05. *P < 0.05, **P < 0.01, ***P < 0.001 for comparisons. All results are represented as mean ± s.e.m.

### Study approval

All experiments were carried out in accordance with Chinese ethical guidelines for the care and use of laboratory animals and approved by the Institute of Neuroscience, Chinese Academy of Sciences.

## Electronic supplementary material

Supplementary Information

## Data Availability

The datasets generated in the current study are available from the corresponding author on reasonable request.

## References

[1] Goda Y, Davis GW (2003). Mechanisms of synapse assembly and disassembly. Neuron.

[2] Waites CL, Craig AM, Garner CC (2005). Mechanisms of vertebrate synaptogenesis. Annu. Rev. Neurosci..

[3] Kutsarova E, Munz M, Ruthazer ES (2017). Rules for shaping neural connections in the developing brain. Front. Neural Circuits.

[4] Niell CM, Smith SJ (2004). Live optical imaging of nervous system development. Annu. Rev. Physiol..

[5] Jontes JD, Emond MR (2012). *In vivo* imaging of synaptogenesis in zebrafish. Cold Spring Harb. Protoc..

[6] Hong JH, Park M (2016). Understanding synaptogenesis and functional connectome in C. elegans by imaging technology. Front. Synaptic Neurosci..

[7] Li Y, Du XF, Liu CS, Wen ZL, Du JL (2012). Reciprocal regulation between resting microglial dynamics and neuronal activity *in vivo*. Dev. Cell.

[8] Niell CM, Smith SJ (2005). Functional imaging reveals rapid development of visual response properties in the zebrafish tectum. Neuron.

[9] Fleisch VC, Neuhauss SC (2006). Visual behavior in zebrafish. Zebrafish.

[10] Meyer MP, Smith SJ (2006). Evidence from *in vivo* imaging that synaptogenesis guides the growth and branching of axonal arbors by two distinct mechanisms. J. Neurosci..

[11] Niell CM, Meyer MP, Smith SJ (2004). *In vivo* imaging of synapse formation on a growing dendritic arbor. Nat Neurosci.

[12] Alsina B, Vu T, Cohen-Cory S (2001). Visualizing synapse formation in arborizing optic axons *in vivo*: dynamics and modulation by BDNF. Nat. Neurosci..

[13] Ruthazer ES, Li J, Cline HT (2006). Stabilization of axon branch dynamics by synaptic maturation. J Neurosci.

[14] Bushati N, Cohen SM (2007). MicroRNA functions. Annu. Rev. Cell Dev. Biol..

[15] Filipowicz W, Bhattacharyya SN, Sonenberg N (2008). Mechanisms of post-transcriptional regulation by microRNAs: are the answers in sight?. Nat. Rev. Genet..

[16] Ye Y, Xu H, Su X, He X (2016). Role of microRNA in governing synaptic plasticity. Neural Plast..

[17] Wayman GA (2008). An activity-regulated microRNA controls dendritic plasticity by down-regulating p250GAP. Proc. Natl. Acad. Sci..

[18] Impey S (2010). An activity-induced microRNA controls dendritic spine formation by regulating Rac1-PAK signaling. Mol. Cell. Neurosci..

[19] Edbauer D (2010). Regulation of synaptic structure and function by FMRP-associated microRNAs miR-125b and miR-132. Neuron.

[20] Hansen KF, Sakamoto K, Wayman GA, Impey S, Obrietan K (2010). Transgenic miR132 alters neuronal spine density and impairs novel object recognition memory. PLoS One.

[21] Remenyi J (2013). miR-132/212 knockout mice reveal roles for these miRNAs in regulating cortical synaptic transmission and plasticity. PLoS One.

[22] Tognini P, Putignano E, Coatti A, Pizzorusso T (2011). Experience-dependent expression of miR-132 regulates ocular dominance plasticity. Nat. Neurosci..

[23] Mellios N (2011). MiR-132, an experience-dependent microRNA, is essential for visual cortex plasticity. Nat. Neurosci..

[24] Kawakami K (2005). Transposon tools and methods in zebrafish. Dev. Dyn..

[25] Xiao T, Roeser T, Staub W, Baier H (2005). A GFP-based genetic screen reveals mutations that disrupt the architecture of the zebrafish retinotectal projection. Development.

[26] Javaherian A, Cline HT (2005). Coordinated motor neuron axon growth and neuromuscular synaptogenesis are promoted by CPG15 *in vivo*. Neuron.

[27] Robles E, Smith SJ, Baier H (2011). Characterization of genetically targeted neuron types in the zebrafish optic tectum. Front. Neural Circuits.

[28] Asakawa K, Kawakami K (2008). Targeted gene expression by the Gal4-UAS system in zebrafish. Dev. Growth Differ..

[29] Stuermer CAO (1988). Retinotopic organization of the developing retinotectal projection in the zebrafish embryo. J. Neurosci..

[30] Hua JY, Smith SJ (2004). Neural activity and the dynamics of central nervous system development. Nat. Neurosci..

[31] Lee S, Stevens CF (2007). General design principle for scalable neural circuits in a vertebrate retina. Proc. Natl. Acad. Sci. USA.

[32] White RM (2008). Transparent adult zebrafish as a tool for *in vivo* transplantation analysis. Cell Stem Cell.

[33] Higashijima S, Masino MA, Mandel G, Fetcho JR (2003). Imaging neuronal activity during zebrafish behavior with a genetically encoded calcium indicator. J. Neurophysiol..

[34] Salta E (2014). A self-organizing miR-132/Ctbp2 circuit regulates bimodal notch signals and glial progenitor fate choice during spinal cord maturation. Dev. Cell.

[35] Xu B (2017). Neurons secrete *miR-132*-containing exosomes to regulate brain vascular integrity. Cell Res..

[36] Kloosterman WP, Lagendijk AK, Ketting RF, Moulton JD, Plasterk RHA (2007). Targeted inhibition of miRNA maturation with morpholinos reveals a role for miR-375 in pancreatic islet development. PLoS Biol..

[37] Wienholds E (2005). MicroRNA expression in zebrafish embryonic development. Science.

[38] Xiao T, Baier H (2007). Lamina-specific axonal projections in the zebrafish tectum require the type IV collagen Dragnet. Nat. Neurosci..

[39] Scott EK (2007). Targeting neural circuitry in zebrafish using GAL4 enhancer trapping. Nat. Methods.

[40] Dreosti E, Odermatt B, Dorostkar MM, Lagnado L (2009). A genetically encoded reporter of synaptic activity *in vivo*. Nat. Methods.

[41] Asakawa K (2008). Genetic dissection of neural circuits by Tol2 transposon-mediated Gal4 gene and enhancer trapping in zebrafish. Proc. Natl. Acad. Sci. USA.

[42] Hua JY, Smear MC, Baier H, Smith SJ (2005). Regulation of axon growth *in vivo* by activity-based competition. Nature.

[43] Ben Fredj N (2010). Synaptic activity and activity-dependent competition regulates axon arbor maturation, growth arrest, and territory in the retinotectal projection. J. Neurosci.

[44] Trachtenberg JT (2002). Long-term *in vivo* imaging of experience-dependent synaptic plasticity in adult cortex. Nature.

[45] Stettler DD, Yamahachi H, Li W, Denk W, Gilbert CD (2006). Axons and synaptic boutons are highly dynamic in adult visual cortex. Neuron.

[46] Hofer SB, Mrsic-Flogel TD, Bonhoeffer T, Hübener M (2009). Experience leaves a lasting structural trace in cortical circuits. Nature.

[47] Xu T (2009). Rapid formation and selective stabilization of synapses for enduring motor memories. Nature.

[48] Yang G, Pan F, Gan WB (2009). Stably maintained dendritic spines are associated with lifelong memories. Nature.

[49] Yang Y (2016). Selective synaptic remodeling of amygdalocortical connections associated with fear memory. Nat. Neurosci..

[50] Fero, K., Yokogawa, T. & Burgess, H. A. The behavioral repertoire of larval zebrafish in *Zebrafish Models in Neurobehavioral Research, Neuromethods* (ed. Kalueff, A.V. & Cachat, J. M.) **52**, 249–291 (2011).

[51] Walsh MK, Lichtman JW (2003). *In vivo* time-lapse imaging of synaptic takeover associated with naturally occurring synapse elimination. Neuron.

[52] McCann CM, Lichtman JW (2008). *In vivo* imaging of presynaptic terminals and postsynaptic sites in the mouse submandibular ganglionitle. Dev. Neurobiol..

[53] Schratt G (2009). MicroRNAs at the synapse. Nat. Rev. Neurosci..

[54] Nakazawa T (2003). p250GAP, a novel brain-enriched GTPase-activating protein for Rho family GTPases, is involved in the *N*-methyl-D-aspartate receptor signaling. Mol. Biol. Cell.

