# Supplementary material for: A transgenic zebrafish model for in vivo long-term imaging of retinotectal synaptogenesis
Source: Sci Rep. 2018 Sep 19;8:14077. doi: 10.1038/s41598-018-32409-y (PMC6145912; doi:10.1038/s41598-018-32409-y)
Supplement: Supplementary file 1 — Supplementary Information [file 41598_2018_32409_MOESM1_ESM.doc]

# Supplementary Information

# A transgenic zebrafish model for *in vivo* long-term imaging of

# retinotectal synaptogenesis

Xu-fei Du^1,*^, Bing Xu^1^, Yu Zhang^1,2^, Min-jia Chen^1,3^ & Jiu-lin Du^1,2,3,*^

^1^ Institute of Neuroscience, State Key Laboratory of Neuroscience, Center for Excellence in Brain Science and Intelligence Technology, Chinese Academy of Sciences, 320 Yue-Yang Road, Shanghai 200031, China

^2^ School of Future Technology, University of Chinese Academy of Sciences, 19A Yu-Quan Road, Beijing 100049, China

^3^ School of Life Science and Technology, ShanghaiTech University, 319 Yue-Yang Road, Shanghai 200031, China

**Supplementary Figure S1. DAPI staining of the retina in *PGUSG* larvae.** (**a**) One optical section of confocal image of the retina with DAPI staining in a 3-dpf *PGUSG* larva. The nasal is upwards, the dorsal is leftwards. (**b**,**c**) Zoom-in and color separate view of the boxed region in (**a**). We chose three sections of each retina to count the number of Sypb-EGFP positive RGCs and DAPI-stained RGCs. Taking DAPI-stained RGCs as total RGCs, we found that 14.5 ± 0.5% (mean ± s.e.m.) of the RGCs were EGFP-positive in *PGUSG* at 3 dpf. As ~ 50% of RGCs could be labelled by the *pou4f3* driver^25^, our *PGUSG* lines can at most label ~ 30% of *pou4f3*-positive RGCs. The calculation was based on data obtained from 3 larvae. Scale bar, 5 μm.

**Supplementary Figure S2.** **Validation of Psd95-DsRedEx as a postsynaptic marker.** (**a**) DsRed (top, red) and SV2 (middle, green) immunostaining of a thin horizontal cryostat section (20 μm in thickness) from a 4-dpf larva with tectal neurons sparsely labelled by Psd95-DsRedEx. M, medial; R, rostral. Scale bar, 5 μm. (**b**) Zoom-in views of three examples of anti-DsRed puncta (yellow arrowheads in **a**) juxtaposing with anti-SV2 puncta. Scale bar, 2 μm. (**c**) Spatial profile of the normalized immunofluorescence intensity of the three juxtapositions along the yellow dotted lines shown in (**b**). (**d**) Summary of the percentage of anti-DsRed puncta juxtaposing with anti-SV2 puncta. The juxtaposition is defined as immunofluorescence intensity overlap > 50%. The data were obtained from 7 cells in 3 larvae.

**Supplementary Figure S3. Example of simultaneous imaging of pre- and postsynaptic sites of retinotectal synapses.** (**a**) First image of a time-lapse series showing Psd95-DsRedEx labelled postsynaptic sites in the dendritic arbor of a tectal neuron (red) and the Sypb-EGFP labelled presynaptic terminals on the axonal arbor of multiple RGCs (green) in a 4-dpf *PGUSG* larva with transient expression of *elavl3:psd95-DsRedEx*. The nasal is upwards. Scale bar, 10 μm. (**b**) Enlarged view of the boxed region in (**a**). Scale bar, 5 μm. (**c**) Enlarged single optical section of the boxed region in (**b**) showing single-channel images and the composite image. The yellow arrowhead (bottom) indicates the association of a presynaptic terminal labelled by Sypb-EGFP (green, top) with a postsynaptic site labelled by Psd95-DsRedEx (red, middle). Scale bar, 5 μm. (**c-i**) 2-h time series at 20 min intervals showing the long-term stable overlap of pre- and postsynaptic labels (yellow arrowhead). Single optical section of the same area as in (**c**) was shown. Time in minutes is indicated in the bottom right corner of each panel.

**Supplementary Figure S4. Knockdown specificity of *miR-132* MO.** Quantification of mature *miR-219*, another brain-enriched miRNA, in 3-dpf zebrafish larvae without MO injection or injected with either control MO or *miR-132* MO by relative quantitative real-time PCR. Data were summarized from six independent experiments. n.s., no significant (One-way ANOVA and Tukey’s multiple comparison test).
